# *SERPINF1* gene variants causing late-onset progressive deforming osteogenesis imperfecta – A study of 18 patients from India

**DOI:** 10.1016/j.bonr.2023.101690

**Published:** 2023-05-26

**Authors:** Agnes Selina, Madhavi Kandagaddala, Vignesh Kumar, Suneetha Susan Cleave Abraham, Sumita Danda, Vrisha Madhuri

**Affiliations:** Christian Medical College, Vellore, India

**Keywords:** Osteogenesis imperfecta, *SERPINF1* gene, Pigment epithelium-derived factor (PEDF) mineralization defect

## Abstract

*SERPINF1* gene variants lead to a severe type of osteogenesis imperfecta (OI) attributed to defects in the matrix mineralization. We present 18 patients with *SERPINF1* gene variants leading to severe progressive deforming OI, the largest series in the world to date. These patients were normal at birth and had the first fracture between 2 months to 9 years; progression of deformities was seen in 12 adolescents who became nonambulatory. Radiologically, compression fractures with kyphoscoliosis, protrusio acetabuli, and lytic lesions in the metaphysis and pelvis were seen in older children with classical popcorn appearance in the distal femoral metaphysis in three. By exome sequencing and targeted sequencing, we identified ten variants. One was unreported and novel; three other novel variants in this series were reported earlier. The recurrent deletion inframe mutation p.phe277del was found in 5 patients from three families. Alkaline phosphatase was elevated in all children on the first visit. Bone mineral density was low in all patients and showed improvement at two years in seven children on regular pamidronate therapy. For others, the 2 year BMD data were not available. The Z scores for four of the seven children showed worsening at the 2-year follow-up.

## 1 Introduction

Osteogenesis imperfecta (OI) is a hereditary bony disorder with reduced bone mass and increased fracture, leading to long bone deformities (Van Dijk and Sillence, 2014). Though the autosomal dominant mutations in the *COL1A1* and *COL1A2* genes cause OI in most patients, many autosomal recessive forms of OI are common in some populations (Forlino and Marini, 2016). One of these, *SERPINF1* (MIM: 613982) (Glorieux et al., 2002), gene variants cause primary defects in endochondral ossification or osteoid mineralization (Marini et al., 2017). OI type VI, associated with *SERPINF1* gene variants, leads to a severe recessive late-presenting form of OI (Glorieux et al., 2002). Patients are healthy at birth with normal sclerae and teeth; fractures usually do not occur till six months of age and are followed by increasing severity, progressive deformities, and loss of mobility by adolescence (Glorieux et al., 2002). Bone histology shows an increased amount of unmineralized osteoid called a ‘fish scale’ lamellar pattern under polarised light. A poor response to bisphosphonate treatment has been reported (Glorieux et al., 2002; Land et al., 2007).

*The SERPINF1* gene is located in chromosome 17p13.3 and encodes the circulating protein pigment epithelium-derived factor (PEDF) (Tombran-Tink et al., 1994). PEDF is a 50 kDa glycoprotein secreted by skeletal progenitors in the bone tissue, which is present in the extracellular matrix and binds to collagen (Tombran-Tink et al., 1994). Loss of PEDF is associated with under-mineralized bones though the exact mechanism is unknown (Becker et al., 2011). The PEDF is a direct inhibitor of angiogenesis by its action on VEGF. In addition, its potent inhibition of angiogenesis is related to its action on osteoclasts, and it is present at the sites of active bone remodelling (Becker et al., 2011; Dawson, 1999). With the loss of PEDF secretion, unrestrained osteoclast differentiation possibly leads to bone resorption. Therefore, the clinical features could resemble osteomalacia with Looser zones, protrusio acetabuli, and deforming OI (Glorieux et al., 2002). Notably, in OI type VI, deformities increase, and fractures decrease with advancing age, suggesting softer rather than brittle bones (Glorieux et al., 2002; Homan et al., 2011).

To date, 97 variants (LOVD database) of *SERPINF1* causing recessive OI have been reported. These are duplications and deletions besides missense and splice-site mutations resulting in loss of PEDF expression. The present study reports 18 patients with *SERPINF1* gene variants in 14 unrelated families describing their phenotype-genotype relation. We report one novel insertion frameshift variant in this largest series reported worldwide.

## 2 Materials and methods

### 2.1 Subjects and their clinical follow-up

All study patients were assessed in the Paediatric Orthopaedics outpatient at a tertiary medical institution receiving OI patients from India, Bangladesh, and Nepal. In a study approved by the institutional review board (IRB min no: 9330 and 10384), we clinically identified 185 patients with osteogenesis imperfecta. They were subjected to mutational analysis. Patients and their parents were given information sheets, and informed consent was obtained before collecting blood samples. Clinical, radiological, and biochemical analyses and bone mineral density (BMD) were performed during their visit. Radiographs were done depending on the need during visits for surgery, pamidronate injections, or when they presented with fractures. Pedigree analysis was done, and blood samples for genetic analysis were collected from the patients and their parents. These identified a cohort of 18 patients from 14 unrelated families with pathogenic variants of the SERPINF1 gene.

### 2.2 Molecular genetic analysis

Eight patients' samples were sent to CENTOGENE AG (Rostock, Germany) for clinical exome sequencing, and the remaining eight patients' blood samples were sent to Medgenome (Bangalore, India) for targeted sequencing. The remaining two samples, belonging to affected family members with identified probands, were done in-house by Sanger sequencing.

### 2.3 Clinical exome sequencing

Blood cards (Centocards, Germany) or extracted genomic DNA of >50 ng were sent to CENTOGENE (Rostock, Germany) for exome sequencing carried out as per the previously published protocol (Madhuri et al., 2020). The pedigree analysis and clinical information on age, sex, height, weight, fracture history, sclera colour, hyperlaxity, dental involvement, joint contractures, and radiological features were used to evaluate the identified variants concerning their pathogenicity, and variants were categorized as 1) pathogenic 2), likely pathogenic and 3) variant of uncertain significance (VUS) according to ACMG guidelines. All significant inheritance patterns were considered. All variants related to the patient's phenotype, except benign or likely benign, were reported. Any relevant variants identified by Next-generation sequencing (NGS) were subjected to Sanger sequenced to exclude NGS artefacts (on behalf of the ACMG Laboratory Quality Assurance Committee 2015).

### 2.4 Targeted sequencing

Genomic DNA was extracted from peripheral blood samples using the QIAamp DNA Blood Mini kit (Qiagen, Hilden, Germany). Isolated DNA was sent to MedGenome Labs (Bangalore, India) for targeted gene capture using a custom capture kit. We designed the gene panel with 18 OI genes (*COL1A1*, *COL1A2*, *IFITM5*, *SERPINF1*, *FKBP10*, *CRTAP*, *PLOD2*, *LEPRE1*, *BMP1*, *SERPINH1*, *CREB3L1*, *TMEM38B*, *SP7*, *WNT1*, *PPIB*, *SPARC*, *MBTPS2* and *TENT5A*) and 17 associated genes (*ADAMTS2*, *COL5A1*, *COL5A2*, *PLOD1*, *MPTPS1*, *MIA3*, *TNFRSF11A*, *TNFRSF11B*, *TNFSF11*, *TNXB*, *SEC24D*, *XYLT2*, *ANO5*, *P4HB*, *LRP5*, *ALPL* and *PLS3*). The libraries were sequenced to mean >80–100× coverage on the Illumina platform. GATK best practices framework for identifying variants in the sample using Sentieon (v201808.01). The sequences obtained were aligned to the human reference genome (GRCh37/hg19) using Sentieon aligner and analyzed using Sentieon to remove duplicates, recalibrate, and realign indels. The Sentieon haplotype caller was used to identify variants relevant to the clinical indication. Gene annotation of the variants was performed using the VEP program against the Ensemble release 91 human gene model. In addition to SNVs and small Indels, copy number variants (CNVs) were detected from targeted sequence data using the Exome Depth (v1.1.10) method. This algorithm detects rare CNVs by comparing the test data's read depths with the matched aggregate reference dataset.

Clinically relevant mutations were annotated using published variants in literature and a set of disease databases - ClinVar, OMIM (updated on 21st November 2018), GWAS, HGMD (v2018.3), and SwissVar. Common variants were filtered based on allele frequency in 1000Genome Phase 3, ExAC (v1.0), gnomAD (v2.1), EVS, dbSNP (v151), 1000 Japanese Genome, and internal Indian population database. The non-synonymous variant effect was calculated using multiple algorithms such as PolyPhen-2, SIFT, MutationTaster2, and LRT. Only non-synonymous and splice site variants found in the osteogenesis imperfecta - OI panel genes were used for clinical interpretation. Silent variations that do not change an amino acid in the coding region were not reported. The variants are classified based on the ACMG guidelines.

### 2.5 Sanger sequencing

After receiving the reports, validation by Sanger sequencing for the proband and their parent samples was done in-house to find the inheritance pattern. Primers were designed to cover the particular variant site using Primer3 software (https://bioinfo.ut.ee/primer3-0.4.0/).

Taq DNA master mix (Ampliqon, Odense, Denmark) was used to amplify the target region by Applied Biosystems®Veriti® Thermal cycler (Thermo Fisher Scientific, USA). PCR products were enzymatically cleaned using ExoSAP IT (Affymetrix, USA), and the sequence reaction was carried out using the BigDye® Terminator v3.1 cycle sequencing kit (Thermo Fisher Scientific, USA). HighPrep™ DTR (MagBioGenomics, USA) magnetic beads-based reagent was used to clean up the Sanger sequencing reaction and read using ABI 3130 genetic analyzer (Applied Biosystems, California, USA). Finally, the obtained nucleotide sequences were analyzed using Mutation Surveyor from Softgenics, BLAST (Basic Local Alignment Search Tool) from NCBI, and BioEdit from Applied Biosystems. The Ensembl genome browser was used to locate cDNA and protein sequences.

### 2.6 Pathogenicity analysis

The pathogenicity of the variants was analyzed using MutationTaster (http://www.mutationtaster.org/), SIFT indel (https://sift.bii.a-star.edu.sg/www/SIFTindels2.html), Loss of Function Tool (LoFtool) accessed through Variant Effect Predictor (VEP), Ensembl (http://asia.ensembl.org/Multi/Tools/VEP?db=core), and MUTpred-Indel (http://mutpredindel.cs.indiana.edu).

Using the experimentally determined three-dimensional structure of PEDF protein (PDB id: 1IMV), any novel variant (p.Lys248_Ala249del) was modelled by submitting the mutated protein sequence to i-Tasser software and analyzed for conformational changes using Swiss PDB viewer (https://spdbv.unil.ch/).

## 3 Results

### 3.1 Clinical findings and radiological findings

The study identified 18 patients (7 males and 11 females), eleven (61 %) from consanguineous parentage. One family had three (Patients 6, 7, and 8), and two had two affected siblings (Patients 4 and 5; Patients 11 and 12). They formed 12.5 % of the examined OI population genetically analyzed from our paediatric orthopaedic outpatient. At the time of presentation, patients were three months to 16 years, and their age at the time of the last visit was between 1 and 23 years. Using the Sillence modified classification, 16 were either OI type IV or III, and two were classed as OI type I. Type I patients included one with only two upper limb fractures and another with two forearm and two proximal femur fractures at the same sites till adolescence. The first fractures in this cohort were documented between 3 months to 9 years, leading to severe long bone deformities over several years. Clinical findings are summarized in Table 1. Five of the patients had light blue sclera, and none had dentinogenesis imperfecta (DI). Deformities were progressive. Upper extremity deformity in 12, and lower extremity deformity was present in 15. Shortening of the lower limbs was seen in all except two children. However, rhizomelia was not noted. Intellectual development appeared normal in all 18 children. Ligamentous laxity was seen in ten children.

**Table 1:** Clinical features (+ present and − absent; NR – not recorded: NA - not applicable).

Family	Patient ID	Sex	Age at first visit (years)	Height at first visit (cm)	Percentile First visit	Age at last visit (years)	Height at last visit (cm)	Percentile last visit	Type	Consanguinity	Family History	Age at first fracture (years)	Total fractures	Blue sclera	Hyperlaxity
I	1	M	9	80	<5	13	153	<75	III	−	−	0.4	>20	+	NR
II	2	F	2	79	<5	5	102	25	III	−	−	1	5	+	−
III	3	F	4	103	<95	7	NR	NA	I	−	−	2	4	−	NR
IV	4	M	10	126	<5	15	133	<5	III	−	+	0.5	20	−	+
	5	F	2	74	<5	7	86	<5	III	−	+	2	2	−	−
V	6	F	14	90	<5	18	95	<5	III	+	+	2	15	−	+
	7	F	12	116	<5	17	120	<5	III	+	+	4	>30	−	+
	8	F	10	127	<5	15	148	<5	I	+	+	2	2	−	−
VI	9	M	11	133	<25	16	150	<5	III	+	−	2	>50	+	+
VII	10	F	4	98	<50	9	114	<5	III	+	−	0.9	8	−	+
VIII	11	M	6	99	<5	10	127	<5	III	+	+	0.7	20	+	NR
	12	F	13	133	<5	15	153	<95	IV	+	+	2	4	−	+
IX	13	F	19	102	<5	25	110	<5	III	+	−	2	20	−	+
X	14	F	18	NR	NA	23	NR	NA	III	+	−	1	>30	−	+
XI	15	M	1	65	<5	4	NR	NA	III	−	−	0.5	12	+	−
XII	16	M	10	102	<5	16	121	<5	III	−	−	0.2	10	−	−
XIII	17	F	13	113	<5	20	NR	NA	III	+	−	0	20	−	+
XIV	18	M	15	129	<5	19	136	<5	III	+	−	9	5	−	+

Percentile: <5 – Short stature; ≥5 and <95 – Normal stature (used CDC height for age percentile 2–20 years).

**Fig. 1:**
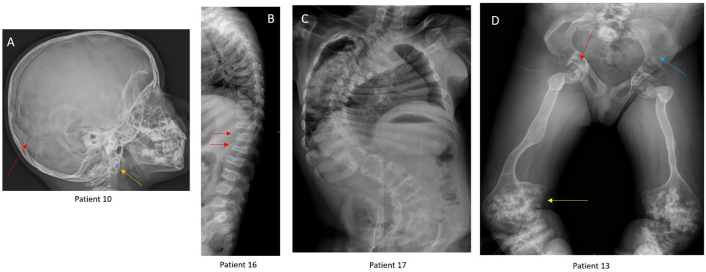
A. Radiographic features of SERPINF1 cohort. Patient (ID 10) lateral radiograph of skull in a four-year-old shows the Wormian bones (red arrow) and basilar invagination (yellow arrow), B. spine radiograph of a 2 year old patient (ID 16) shows multiple vertebral compression fractures, C. A 15 year old patient (ID 17) shows scoliosis, D. a 16-year-old (ID 13) patient AP view of pelvis and both femur shows protrusio acetabuli (red arrow), popcorn calcification in the epiphysis (yellow arrow) and lytic lesions (blue arrow) in the pelvic bones.

The radiological findings are presented in Table 2. Wormian bones were present in two children. Basilar invagination was seen in one child. Vertebral compression fractures were evident in 13 (72.2 %), all above five years. Scoliosis was seen in ten patients, all above ten years of age. Upper extremity intramedullary rodding was required for only one of our patients (5.5 %), whereas intramedullary rodding of the lower extremity was done for eleven (61.1 %). Protrusio acetabuli was present in eight patients in the above-ten age group. The lytic lesions in the metaphysis and pelvis were seen in two young adult patients (Patients 13 and 14). The popcorn appearance of the bulbous distal metaphyses was seen in older patients with severe deformities (Patients 6, 13, and 14). Hypertrophic callus was seen in two toddlers (Patients 2 and 15). While the fractures were frequent in children under ten, older children predominantly had deformities. The radiological appearance is shown in Fig. 1.

**Table 2:** Radiological features, biochemical values, and bone mineral density. NA - not available; R denoted repeated value.

Family	ID	Bowing of upper extremities	Bowing of lower extremities	Lower extremities Intramedullary rodding	Vertebral compression fractures	Scoliosis	Kyphosis	Acetabuli protrusio	Other features	Age at first ALP (years)	S. ALP at first visit U/L	Age at last ALP taken (years)	S. ALP at last visit U/L	Total cycles of pamidronate	BMD (lumbar) g/cm^2^	Z score	BMD (lumbar) After 2 years g/cm^2^	Z score After 2 years
I	1	+	+	+	NA	+	+	No	−	9	372	13	330	3	NA	NA	NA	NA
II	2	−	+	−	Present	No	No	No	Exuberant callus formation and wide canals	NA	NA	NA	NA	12	0.284	NA	0.723	NA
III	3	−	+	−	Multiple	No	No	No	−	4	277	NA	NA	2	NA	NA	NA	NA
IV	4	+	+	+	Present	NA	+	No	−	8	252	9	254	6	0.514	−1.8	NA	NA
	5	+	+	−	Multiple	NA	No	No	−	2	204	NA	NA	4	0.327	−3.3	NA	NA
V	6	+	+	−	Multiple	+	+	Yes	Popcorn calcification	6	549	18	301	12	0.345	−7.7	0.413	−9.0
	7	+	+	+	Multiple	+	+	Yes	−	5	549	17	137	11	0.605	−1.9	0.762	−2.7
	8	−	−	−	NA	NA	NA	No	−	3	177	15	186	8	0.693	−1	0.835	−1.3
VI	9	+	+	+	NA	NA	NA	No	−	5	292	8	211	16	0.655	0.4	0.795	0.5
VII	10	+	+	+	Multiple	+	No	No	−	4	264	NA	NA	3	NA	NA	0.45	−2.1
VIII	11	+	+	+	Present	+	+	No	−	6	306	11	275	17	0.327	NA	0.201	NA
	12	−	−	+	NA	NA	NA	Yes	−	13	305	15	111	2	0.363	NA	0.769	NA
IX	13	+	+	−	Multiple	+	+	Yes	Lytic lesion present popcorn calcification	10	150	NA	NA	11	0.85	−1.6	NA	NA
X	14	+	+	+	Multiple	+	+	Yes	Lytic lesion present popcorn calcification	11	1367-R	NA	NA	2	0.257	−9.1	NA	NA
XI	15	+	+	−	NA	NA	NA	No	Exuberant callus formation and wide canals	1	392	NA	NA	1	NA	NA	NA	NA
XII	16	+	+	+	Multiple	+	+	yes	−	1	261	16	161	37	0.347	NA	0.617	−1.2
XIII	17	+	+	+	Multiple	+	+	Yes	−	NA	NA	NA	NA	6	NA	NA	NA	NA
XIV	18	−	+	+	Multiple	+	NA	Yes	−	15.5	430	16	286	2	0.219	−6.1	NA	NA

Serum alkaline phosphatase (S.Alp): Normal range < 350 U/L.

Table 2 provides BMD, alkaline phosphatase at the first and last follow-up, and pamidronate cycles administered in the intervening period. Except for six children, most children had irregular treatment and follow-up. The BMD values were low at the first visit in all patients. The values showed an increase in 6 children two years after pamidronate treatment; however, all except one showed worsening of lumbar spine Z scores, with Z scores varying from 0.4 to −7.7 at the beginning of the treatment and 0.5 to −9 two years after pamidronate therapy. Two adolescents from the twelve nonambulatory patients became independent ambulators after pamidronate therapy and surgery. Serum calcium levels were normal, whereas the serum alkaline phosphatase levels were elevated for all the patients at the first visit. Two children continued to have severe pain and showed no improvement despite regular pamidronate therapy (Patients 6 and 16). They were started on Denosumab and showed improvement in pain after the first dose. The remaining parameters of them will be reassessed after one year.

### 3.2 Genetic analysis

We identified ten variants (two nonsense variants, one missense variant (VUS), two frameshift variants, four in-frame deletions, and one in-frame duplication) in 18 patients from 14 unrelated families (Table 3). A novel variant c.722_723insC (p.Met243Hisfs*6) resulted in a single base pair insertion at exon 6, leading to a frameshift and premature truncation of the protein 6 amino acid downstream to the codon 243. Six variants c.829_831del, c.907C>T, c.838_839del, c.621_623del, c.742_747del, and c.871_873del reported earlier by us are also included in this series.

**Table 3:** Genetic analysis of the 18 patients (NA - not available) VUS - variant of uncertain significance; hetero-heterozygous; homo-homozygous.

Family	Patient ID	Exon	c.DNA	Protein change	Variant Type	Type	Zygosity	Reported/novel	Family segregation analysis
I	1	Exon 6	c.722_723insC	p.Met243Hisfs*6	Frameshift	VUS	Homo	Novel	NA
II	2	Exon 7	c.871_873del	p.Glu291del	In-frame	Pathogenic	Homo	Reported (Madhuri et al., 2020)	Parents-hetero
III	3	Exon 5	c.621_623del	p.Leu208del	In-frame	VUS	Hetero (carrier state)	Reported (Madhuri et al., 2020)	Father-hetero, mother-wildtype
IV	4	Exon 6	c.742_747del	p.Lys248_Ala249del	In-frame	Likely pathogenic	Homo	Reported (Madhuri et al., 2020)	Parents-hetero, sister-homo
	5	Exon 6	c.742_747del	p.Lys248_Ala249del	In-frame	Likely pathogenic	Homo	Reported (Madhuri et al., 2020)	Parents-hetero, brother-homo
V	6	Exon 7	c.829_831del	p.Phe277del	In-frame	Pathogenic	Homo	Reported (Rauch et al., 2012)	Parents-hetero, two sisters-homo, youngest-wildtype
	7	Exon 7	c.829_831del	p.Phe277del	In-frame	Pathogenic	Homo	Reported (Rauch et al., 2012)	Parents-hetero, two sisters-homo, youngest-wildtype
	8	Exon 7	c.829_831del	p.Phe277del	In-frame	Pathogenic	Homo	Reported (Rauch et al., 2012)	Parents-hetero, two sisters-homo, youngest-wildtype
VI	9	Exon 7	c.829_831del	p.Phe277del	In-frame	Pathogenic	Homo	Reported (Rauch et al., 2012)	Father-hetero and mother-hetero
VII	10	Exon 7	c.829_831del	p.Phe277del	In-frame	Pathogenic	Homo	Reported (Rauch et al., 2012)	Parents-hetero
VIII	11	Exon 7	c.907C>T	p.Arg303*	Nonsense	Likely Pathogenic	Homo	Reported (Clin Var SCV001976526)	Parents-hetero, sister-homo
	12	Exon 7	c.907C>T	p.Arg303*	Nonsense	Likely Pathogenic	Homo	Reported (Clin Var SCV001976526)	Parents-hetero, brother-homo
IX	13	Exon 7	c.907C>T	p.Arg303*	Nonsense	Likely Pathogenic	Homo	Reported (Clin Var SCV001976526)	Mother-hetero, father NA
X	14	Exon 7	c.838_839delCT	p.Leu280Glufs*20	Frameshift	Pathogenic	Homo	Reported (Mrosk et al., 2018)	NA
XI	15	Exon 7	c.838_839delCT	p.Leu280Glufs*20	Frameshift	Pathogenic	Homo	Reported (Mrosk et al., 2018)	Parents-hetero
XII	16	Exon 3	c.205C>T	p.Arg69*	Nonsense	Pathogenic	Homo	Reported (Clin Var SCV 0017555272)	Parents-hetero
XIII	17	Exon 8	c.1226T>C	p.Ile409Thr	Missense	Pathogenic	Hetero (carrier state)	Reported (ClinVar SCV000400901)	NA
XIV	18	Exon 3	c.259_260ins	p.Ala91_Ser93dup	In-frame	VUS	Homo	Reported (Tucker et al., 2012)	Parents-hetero

A single heterozygous variant of a known pathogenic mutation reported in the ClinVar database (also heterozygous) was seen in one patient (patient 17) with a severe phenotype. We found a deletion in-frame c.829_831del p.(Phe277del) in three patients from one family and two from two unrelated families, all from the same region.

Two siblings (patients 11 and 12) with varying phenotypes carried a previously reported homozygous nonsense variant c.907C>Tp. (Arg303*) creating a premature stop codon. For all the identified homozygous variants, parental carrier testing showed heterozygosity.

### 3.3 Evaluation of the novel variants

The four novel variants from this population, including the three previously reported briefly from the institution, were evaluated, and the pathogenicity scores are summarized in Table 4. The results indicate that the variants are pathogenic in their effects on the PEDF protein. The Mutpred-Indel software returns a general score indicating the probability of the frameshifting variant being pathogenic. In our study, all the variants are scored above 0.50, suggesting pathogenicity. The LoFtool provides a gene intolerance score based on loss-of-function variants; the low score (<0.283) in our study for *SERPINF1* variants indicates that the gene is intolerant to variations.

**Table 4:** *In silico* analysis of novel variants identified in the *SERPINF1* gene.

Family	Patient ID	Variants	MutationTaster	SIFT indel	LoFtool	MUTpred indel
I	1	c.722_723insC p.(Met243Hisfs*6)	Disease-causing	Damaging	0.283-probably damaging	0.58123
II	2	c.871_873del p.(Glu291del)	Disease-causing	Damaging	0.283-probably damaging	0.87265
III	3	c.621_623del p.(Leu208del)	Disease-causing	Damaging	0.283-probably damaging	0.89903
IV	4 and 5	c.742_747del p.(Lys248_Ala249del)	Polymorphism	Damaging	0.283-probably damaging	0.86461

All the amino acids mutated in our patients were visualized in the experimentally determined three-dimensional structure of the PEDF protein retrieved from the PDB (Id:1IMV). It was observed that Lys248 was part of a loop, and Ala249 was part of a β-sheet. The reactive site amino acid residues (GAGTTPSPGLQQPAHL) (Wright, 1996) are part of a loop structure and were proximal to the Lysine and Alanine residues. The amino acids that were deleted in the 3rd (Leu208) and 4th patients (Glu291), are part of a β-sheet and an α-helix, respectively.

**Fig. 2:**
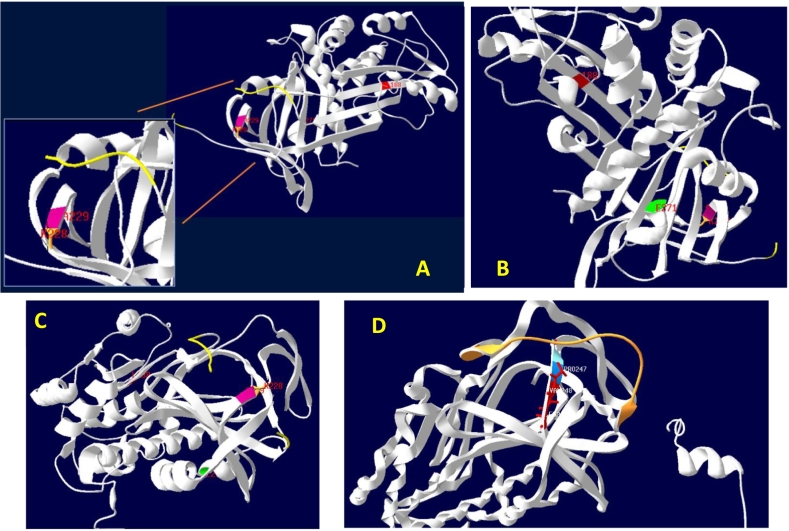
Three-dimensional structure of wildtype SERPINF1 structure in Figs. A, B, and C showing the location of amino acids mutated in our patients. Fig. A shows the following mutated sites - Lys248 (orange loop structure), Ala249 (pink β sheet structure), and Leu 208 (red β sheet) (An inset focusing on the orange, yellow and pink residues); Fig. B shows the mutation at the proximal site- Glu291 (green α helix); Fig. C shows wildtype conformation of Reactive loop (yellow) and Lysine (orange) and alanine (pink) The thread like reactive loop amino acid residues are highlighted in yellow in A, B C and D; Fig. D shows the mutated SERPINF1 (patients 4 and 5) formation of a single β sheet due to the deletion of the two amino acids lysine and alanine. The amino acids before Proline 246(Proline 246 in dark blue) is coloured light blue and the one after (valine 248) is shown in red. The deleted amino acids are lysine and alanine. The reactive loop is marked in yellow.

A variant PEDF protein structure with Lys248_Ala249del, modelled using i-Tasser, had a confidence score (C-score) of 0.82 and showed 98.7 % identity with the native protein structure 1IMV. The following structural changes were observed. The original β-sheet-loop-β-sheet was replaced with a single β-sheet. The orientation of the reactive loop has changed with reference to the wild-type conformation (Fig. 2).

## 4 Discussion

Eight patients with type VI OI were first described in 2002 based on the ‘fish scale appearance’ findings in the bone biopsies available (4). Subsequently, the genetic identification of *SERPINF1* variants showed it to form 6 % of the OI population (Glorieux et al., 2002; Land et al., 2007). The studies report small case series from different populations (Cho et al., 2013; Glorieux et al., 2002; Homan et al., 2011; Minillo et al., 2014). In our large population study, 18 *SERPINF1* cases with ten variants formed 12.5 % of the OI population from the Indian subcontinent. The higher percentage is possibly because of greater consanguinity in this population (Stephen et al., 2015). In the described phenotype, white or faintly blue sclerae may be present with absent DI (Glorieux et al., 2002); in our series, five patients had light blue sclera and no DI. Type VI shows moderate to high severity, appearing late between the ages of three months to two years, with evidence of increased bone turnover in the form of raised alkaline phosphatase, progressive involvement, and, eventually, long bone deformities (Glorieux et al., 2002; Homan et al., 2011). Our population showed similar progression, with the first fractures presenting from 3 months to 9 years, the earliest instance associated with manipulation for a clubfoot deformity; the most likely age for the first fracture to occur was around two years. Though there is no existing evidence on the cause for late onset fracture. It is possible that increased muscle forces and weight can cause fractures after 2 years. Another possible reason could be the normal PEDF levels during birth which could decrease when children grow. However, there is no evidence for the latter. The OI form appeared mild at first, but gradually the fracture rate increased in older childhood, and the deformities progressed, with almost all having long bone and spinal deformities by their teenage years even though the fracture frequency had decreased. These trends are similar to the experience noted by other studies (Becker et al., 2011; Glorieux et al., 2002; Hayat et al., 2020).

Radiological features seen were similar to those described previously. The long bones with wider medullary canals than classical OI but thin cortices were seen (Minillo et al., 2014; Zhang et al., 2018), bulbous metaphyses, and a lack of trabecular appearance suggesting severe osteoporosis (Glorieux et al., 2002; Hayat et al., 2020). All except one of the older children showed vertebral collapse (Glorieux et al., 2002; Homan et al., 2011). Good callus formation in two acute fractures suggests that the formation of immature bone was unaffected. Three children showed classical popcorn densities in the lower femur, and another two showed metaphyseal lytic changes with multiple lytic lesions in the pelvis and metaphyses (Glorieux et al., 2002). Changes of Osteomalacia were present on radiographs in older individuals above 15 years, such as protrusion acetabuli and bowed bones (suggestive of osteomalacia), but no widening of the physis or other rachitic changes (Glorieux et al., 2002; Homan et al., 2011; Minillo et al., 2014). The osteomalacic manifestations such as protrusio are not often seen in the other forms of OI.

Progression of various clinical manifestations is seen in all series, even when on treatment (Land et al., 2007). Seven of our children had lost mobility. Two were bedridden and unable to sit up; the other five were in wheelchairs. Our observation is similar to the findings of Glorieux et al., where 50 % had wheelchair mobility (Glorieux et al., 2002). Despite ten children being rodded for lower limb deformity in this group, only two became ambulators.

*SERPINF1* gene at 12.5 % is the most prevalent recessive gene isolated so far from our population and is the cause of significant deformities and disability. This high incidence of the *SERPINF1* variant is also noted in the Chinese population (Li et al., 2020). *SERPINF1* encoding the PEDF induces the expression of osteoprotegerin (OPG), a physiological inhibitor of osteoclastogenesis, through the blockade of RANKL (Akiyama et al., 2010). The loss of function mutation in *SERPINF1* leads to increased activation and differentiation of the osteoclasts mediated by the misregulated RANKL/OPG system, thus causing bone mass degradation (Germain-Lee, 2011). If these cases are picked up early because of their classical radiological appearance, the early treatment with bisphosphonate is expected to be rewarding. Bisphosphonates, however, are known to be less effective for type VI OI (Land et al., 2007). The reason might be that the increased amount of unmineralized osteoid impairs the ability of bisphosphonates to attach, thus reducing their toxicity for osteoclasts. In the studies by Land et al. and Cho et al., intravenous pamidronate therapy improved bone mineral density, but a gain in mobility score and a reduction in fracture incidence was less (Cho et al., 2013; Land et al., 2007). In the study by Becker et al., vertebral reshaping and increased vertebral height were reported (Becker et al., 2011). In the present study, bone mineral density increase was seen in seven patients who received regular bisphosphonate therapy for several years, reducing pain but not improving mobility. The study children initially showed high serum alkaline phosphatase, which normalized when the patients received bisphosphonate therapy. A better response with Denosumab has been reported (Semler et al., 2012), and this was resorted to for unresponsive cases in our group and showed early pain improvement.

We found ten variants in the *SERPINF1* gene in our study. Most are in-frame deletions and have been previously reported from all populations. We found four novel variants; three were in-frame deletions which we reported in an earlier population study (Madhuri et al., 2020). The fourth and previously unreported is an insertion frameshift variant c.722_723insC (p.Met243Hisfs*6).

We found the recessive trait in all except two of our patients, with 11/16 of these patients showing consanguinity. The two with the heterozygous presentation but with severe classical phenotypes were possibly due to missing out on a second variant like a large deletion, duplication, or deep intron variant. Rauch et al., 2012 previously reported one patient with a single heterozygous pathogenic variant (Rauch et al., 2012). One of the two heterozygous patients was a deletion variant we reported earlier (Madhuri et al., 2020). The other missense variant, c.1226T>C, is reported earlier as pathogenic in the Clin Var database in the homozygous state.

Five of our patients with c.829_831del p.(Phe277del) in-frame deletion variant from three families came from neighboring locations. This pathogenic variant, previously described by Rauch et al. in a Nepalese child, had the first fracture at six months (Rauch et al., 2012). Later, Stephen et al. reported it in a proband of Indian origin with three affected brothers (Stephen et al., 2015). In our series, the above variant showed varying presentations. In a single family, the severest involvement was a bedridden child with multiple fractures and severe pulmonary compromise starting at a young age. At the same time, the second sibling was comfortable but wheelchair-bound at the same age, while the third child was independently mobile even after adolescence, with just two upper limb fractures during her lifetime. The wide variation of phenotypes in the siblings suggested epigenetic variations or modifiers genes countering the deleterious effect of this gene. This mutation, now reported from 5 of our patients and twice from other studies from the region earlier, should be considered the recurrent *SERPINF1* mutation in our region and the founder mutation in South India.

We found a nonsense variant c.907C>Tp. (Arg303*)which is likely pathogenic, causing two different phenotypes in siblings. The Sister had a type IV phenotype with four fractures, whereas the brother had a type III phenotype with twenty fractures and was bedridden. The brother underwent intramedullary rodding of the lower extremities. The same variant was found in another girl from a different family. She was severely deformed with multiple vertebral compression fractures, scoliosis, kyphosis, and upper and lower limb deformity.

*SERPINF1* belongs to inhibitory serpins, which comprise alpha-helix and beta-strands and an external reactive centre loop (RCL) (Huntington, 2011). The RCL contains the active site recognized by the target proteases. During their inhibitory action, the serpins undergo a rapid conformational change that traps the protease in a covalent complex (Huntington, 2011). The Lys248_Ala249 del variant in two siblings in our study resulted in the formation of a single β sheet replacing the original β-sheet-loop-β-sheet. These structural changes may affect the protein's flexibility to undergo a conformational change, requiring further proteomic studies to confirm the structural effects.

The major limitation of this study was the lack of data on serum PEDF levels and incomplete follow-up data because of irregular treatment in some cases.

In conclusion, *SERPINF1* variants cause a mineralization defect resulting in an OI, with a combination of congenital osteoporosis and osteomalacia caused by increased osteoclast activity, which forms 12.5 % of our patients. The founder variant in our population is c.829_831del. A widely varying phenotype and normal birth history are seen. Late presentation with progression into adulthood with deformities being the primary feature at an older age, combined with osteomalacic features of looser zones, protrusion acetabuli, and hypermineralization at the physes, epiphyseal stippling, and lytic bone changes should alert the clinician to its possibility.

## CRediT authorship contribution statement

**Vrisha Madhuri:** Conceptualization, Formal analysis, Funding acquisition, Investigation, Supervision, Validation, Writing – original draft, Writing – review & editing. **Agnes Selina:** Data curation, Formal analysis, Methodology, Writing – original draft, Writing – review & editing. **Madhavi Kandagaddala:** Data curation, Formal analysis, Visualization, Writing – review & editing. **Vignesh Kumar:** Data curation, Formal analysis, Methodology. **Suneetha Susan Cleave Abraham:** Data curation, Formal analysis, Visualization. **Sumita Danda:** Writing - review & editing.

## Declaration of competing interest

All authors Agnes Selina, Madhavi Kandagaddala, Vignesh Kumar, Suneetha Susan Cleave Abraham, Sumita Danda and Vrisha Madhuri declare no conflict of interest.

## Data Availability

Data will be made available on request.
